# Endoplasmic Reticulum Stress and Unfolded Protein Response in Neurodegenerative Diseases

**DOI:** 10.3390/ijms21176127

**Published:** 2020-08-25

**Authors:** Rose Ghemrawi, Mostafa Khair

**Affiliations:** 1 College of Pharmacy, Al Ain University, Abu Dhabi 112612, UAE; 2 Core Technology Platforms, New York University Abu Dhabi, Abu Dhabi 129188, UAE; mrk6@nyu.edu

**Keywords:** ER stress, unfolded protein response, neurodegeneration

## Abstract

The endoplasmic reticulum (ER) is an important organelle involved in protein quality control and cellular homeostasis. The accumulation of unfolded proteins leads to an ER stress, followed by an adaptive response via the activation of the unfolded protein response (UPR), PKR-like ER kinase (PERK), inositol-requiring transmembrane kinase/endoribonuclease 1α (IRE1α) and activating transcription factor 6 (ATF6) pathways. However, prolonged cell stress activates apoptosis signaling leading to cell death. Neuronal cells are particularly sensitive to protein misfolding, consequently ER and UPR dysfunctions were found to be involved in many neurodegenerative diseases including Alzheimer’s disease, Parkinson’s disease, amyotrophic lateral sclerosis and prions diseases, among others characterized by the accumulation and aggregation of misfolded proteins. Pharmacological UPR modulation in affected tissues may contribute to the treatment and prevention of neurodegeneration. The association between ER stress, UPR and neuropathology is well established. In this review, we provide up-to-date evidence of UPR activation in neurodegenerative disorders followed by therapeutic strategies targeting the UPR and ameliorating the toxic effects of protein unfolding and aggregation.

## 1. ER Functions and Connections with Other Organelles

The endoplasmic reticulum (ER) is a large organelle spread throughout the cytoplasm and divided into three morphologies that include the nuclear envelope (NE), peripheral ER cisternae, and an interconnected tubular network [1]. ER is considered multifunctional since it is specialized in lipid and steroid synthesis, Ca^2+^ homeostasis and storage, carbohydrate metabolism, and protein synthesis [2]. This multi-functional nature requires, in addition to a myriad of proteins and a unique physical structure, interconnections and coordination with other organelles. Thus, ER has numerous contact sites with all membrane-bound organelles, including the plasma membrane (PM), mitochondria, Golgi, endosomes, and peroxisomes [1]. Structural connectivity at the level of ER-PM and ER-mitochondria junctions is the best studied.

### 1.1. ER and Plasma Membrane

The ER is the cell’s major Ca^2+^ store and forms an extensive and dynamic network of contacts with the PM. It is becoming increasingly evident that contact sites between the ER and the PM play a role in Ca^2+^ exchange. Influx of Ca^2+^ across the plasma membrane in many cells is achieved by store operated Ca^2+^ entry (SOCE). At ER-PM junctions, stromal-interacting molecule (STIM) proteins sense a drop in ER Ca^2+^ levels, undergo a conformational change repositioning tubular structures throughout the ER to ER-PM and directly activate Orai, the pore-forming component of the Ca^2+^- release-activated Ca^2+^(CRAC) channel, triggering channel opening and Ca^2+^ influx [3]. In addition, ER-PM contact sites are important for the phosphatidylinositol metabolism specifically for the regulation of the lipid signaling molecule phosphatidylinositol 4-phosphate (PI4P). This is controlled by the oxysterol-binding homology (Osh) protein family and the integral ER membrane proteins vesicle-associated membrane protein- associated protein (VAP) after the activation of Sac1 phosphatase [1]. Deletion of Osh proteins in yeast cells resulted in a six to seven-fold increase in PI4P levels; furthermore, the addition of recombinant Osh3 to a microsome fraction depleted peripherally bound proteins able to stimulate Sac1 phosphatase activity, suggesting that Osh proteins control PI4P levels at ER-PM contact sites [4].

### 1.2. ER and Mitochondria

The sites of ER in contact with mitochondria have been referred to as mitochondria-associated ER membrane (MAM). This communication is vital for the cell fate; it operates as a structural allocation for multiple scaffold proteins and regulatory factors and it is associated with multiple functions including lipid transfer, autophagosome formation, mitochondrial fission, Ca^2+^ homeostasis and apoptosis [5]. At ER contact sites, there is an influx of Ca^2+^ into the intermembrane space and matrix of the mitochondria. ER channels release Ca^2+^ directly to the mitochondrial membrane and this requires IP3 receptor (IP3R) interaction with the voltage-dependent anion selective channel protein 1 (VDAC1). Changes in Ca^2+^ levels have been shown to affect apoptosis, mitochondrial division and motility, and to regulate the activity of mitochondrial Ca^2+^-binding proteins [1,6]. MAM is also necessary for lipid flipping during lipid biosynthesis. During this process, phosphatidylserine (PS) is synthesized from phosphatidylalanine on the ER membrane; then, PS conversion to phosphatidylethanolamine (PE) uses proteins on the mitochondria and PE conversion to phosphatidylcholine (PC) uses ER localized enzymes. Therefore, the phospholipid moves between the two membranes before each conversion step. Furthermore, ER tubules were shown to be involved in mitochondrial biogenesis by defining the position of the mitochondrial division machinery recruitment in yeast and mammalian cells [7]. Several types of molecular bridges mediate the contacts between these two organelles, such as the ER-mitochondria encounter structures (ERMES) complex identified in yeast and the mitochondrial fusion protein mitofusin 2 (Mfn2) in mammalian cells, which suggest that contact site formation may be highly regulated during different ER-mitochondria functions [7]. Alterations in ER-mitochondria signaling have pleiotropic effects on a variety of intracellular events resulting in mitochondrial damage, Ca^2+^ dyshomeostasis, ER stress, defects in lipid metabolism, autophagy, reduced respiratory chain activity and oxidative phosphorylation; mitochondria and ER play a central role in the regulation of neurological activities. Multiple studies revealed that these alterations are common in neurodegenerative disease [8,9].

## 2. ER Stress and the Unfolded Protein Response (UPR)

The ER is the principal site for biosynthesis of proteins, post-translational modification, folding and assembly of newly synthesized proteins. As a membranous compartment, the ER is extremely sensitive to changes that affect its structure, integrity and function leading to a disruption of proteins’ folding. If the final tertiary structure cannot be attained, unfolded proteins are translocated to the cytosol and subjected to ubiquitination and proteasome-dependent degradation, known as ER-associated degradation (ERAD). The accumulation of unfolded or misfolded proteins inside the cell results in the failure of the ER to cope with the excess of protein load leading to “ER stress” and to several pathological conditions [10].

Eukaryotic cells can adapt by attenuating the rate of protein synthesis, upregulating the expression of genes encoding chaperones and other proteins that prevent polypeptide aggregation and degrading accumulated misfolded proteins. This set of cellular responses is obtained after the activation of an integrated intracellular signaling cascade: the “Unfolded Protein Response” (UPR).

**Figure 1:**
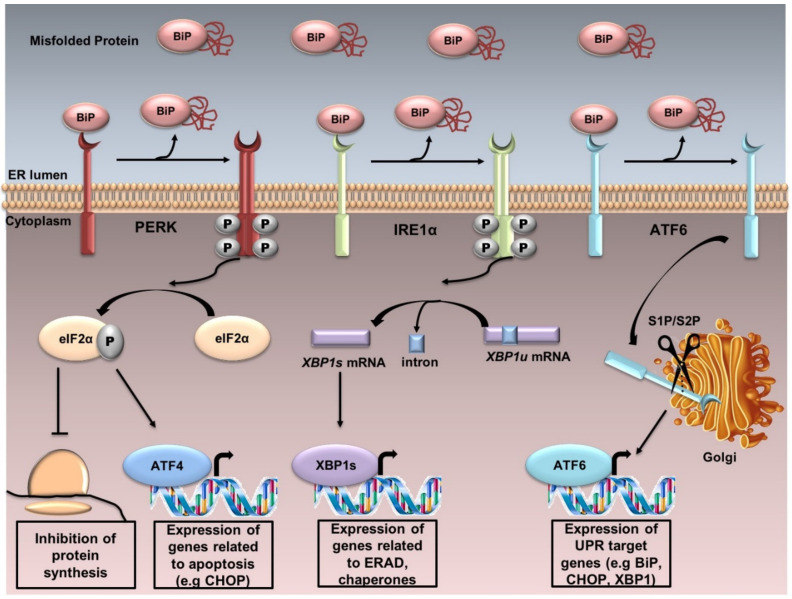
The UPR. UPR is controlled by three sensor proteins: PERK, IRE1α and ATF6. Under normal conditions, these proteins are associated with BiP and thus remain inactive. Under ER stress, BiP is released from these sensor proteins, and UPR cascade is activated after further dimerization and autophosphorylation of PERK and IRE1α, and regulated intramembrane proteolysis of ATF6. PERK activation induces the phosphorylation of eIF2α. Phosphorylated eIF2α reduces the global protein synthesis and promotes the translation of the activating transcription factor 4 (ATF4) which upregulates the genes related to apoptosis. IRE1α dimerizes, autotransphosphorylates, and its endoribonuclease activity enables the splicing of the unspliced X-box binding protein 1 XBP1u to the spliced XBP1s. XBP1s upregulates the transcription of several genes involved in UPR and ERAD. ATF6 is localized at the ER in unstressed cells. Under ER stress, ATF6 translocates from the ER to the Golgi, where it is cleaved sequentially by the enzymes site 1 protease (S1P) and site 2 protease (S2P). Active ATF6 is transferred into the nucleus, where it binds the promoters of several genes involved in UPR (such as C/EBPα-homologous protein (GADD153 or CHOP), BiP, XBP1).

UPR is controlled by three sensor proteins: PERK (PKR-like ER kinase), IRE1α (inositol-requiring transmembrane kinase/endoribonuclease 1α) and ATF6 (activating transcription factor 6). Under normal conditions, these proteins are associated with BiP or GRP78 (78 KDa glucose-regulated protein) and thus remain inactive. Although the mechanism behind the detection of the unfolded protein accumulation in the ER by PERK and IRE1α remains obscure, Carrara et al. [11] proposed that the ATPase domain of BiP interacts with PERK an IRE1α, which dissociates when an unfolded protein binds to the canonical substrate binding domain of BiP. Therefore, under ER stress, BiP is released, and UPR cascade is activated after further dimerization and autophosphorylation of PERK and IRE1α, as well as regulated intramembrane proteolysis of ATF6 (Figure 1).

PERK is a type I ER transmembrane protein, containing a cytoplasmic serine/threonine protein kinase domain. PERK activation induces the phosphorylation of the α subunit of eukaryotic translation initiation factor (eIF2α) at the residue serine 51. Phosphorylated eIF2α (p-eIF2α) reduces the global protein synthesis and promotes the translation of ATF4 because of a longer resident time on its first uORF (upstream open reading frame) of the translation initiation complex. ATF4 upregulates the genes related to apoptosis, including the pro-apoptotic factor GADD153 or CHOP. The PERK/ATF4/CHOP signaling pathway plays a pivotal function in inducing cell apoptosis; PERK^−/−^, ATF4^−/−^ cells and eIF2α (Ser51Ala)^−/−^ cells fail to induce CHOP during ER stress [12].

IRE1 is also a type I ER transmembrane protein which senses ER stress and has serine/threonine kinase and endonuclease activities. The mammalian genome encodes for two isoforms: IRE1α and IRE1β [13]. Most mammalian UPR research is conducted by IRE1α. The accumulation of unfolded proteins in the ER stimulates IRE1α oligomerization and autophosphorylation, activating the endoribonuclease activity enabling the splicing of *Xbp1* mRNA through the cleavage of a 26-nucleotide sequence. This results in a frame shift of the unspliced form (XBP1u), producing eventually an active transcription factor, the spliced form (XBP1s). XBP1s upregulates the transcription of several genes involved in UPR and ERAD. Under prolonged stress, IRE1α promotes cell death by activating the apoptotic-signaling kinase-1 (ASK1), followed by the activation of downstream kinases, Jun-N-terminal kinase (JNK) and p38 mitogen-activated protein kinase (p38 MAPK), leading to apoptosis [14]. Furthermore, IRE1 RNase activity is involved in the regulated IRE1-dependent decay (RIDD), known to selectively degrade ER-associated mRNAs coding secretory or membrane proteins in order to unburden the protein load of the ER [15,16].

ATF6 is the third transmembrane mediator of UPR. It is an ER-associated type II transmembrane protein. Two isoforms have been described: ATF6α and ATF6β [17]. ATF6α has a higher transcriptional activity than ATF6β. Under ER stress, ATF6 translocates from the ER to the Golgi, where it is cleaved sequentially by S1P and S2P. Active ATF6 is transferred into the nucleus, where it binds the promoters of several genes involved in UPR (such as CHOP, BiP, XBP1) and induces target-gene transcription [18].

Overall, UPR mediators are involved in maintaining ER homeostasis. Excessive or long-term accumulation of misfolded proteins leads to the failure of UPR’s adaptive responses and the initiation of the regulated cell death.

### 2.1. Interaction between UPR, Protein Aggregation and Neurodegeneration

**Figure 2:**
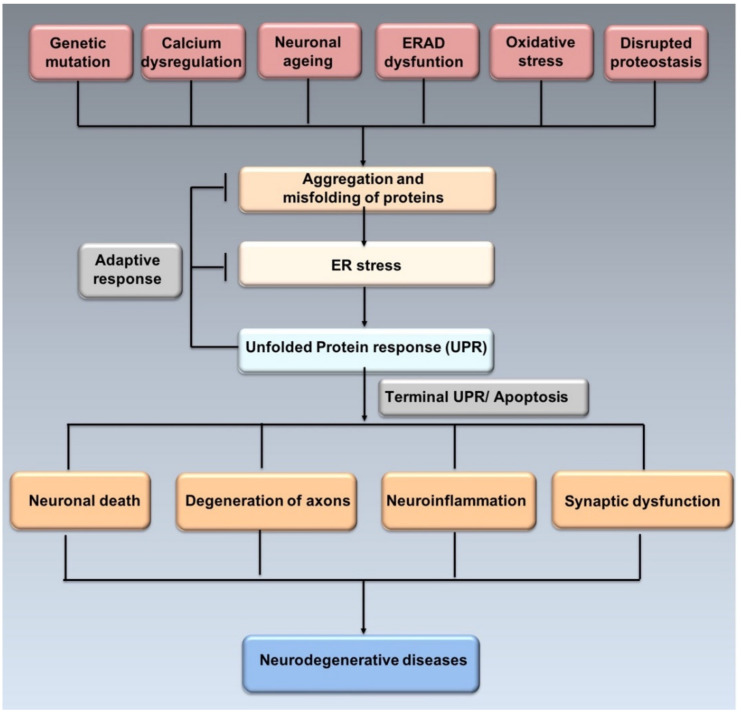
ER stress, UPR and neurodegeneration. Genetic mutations, ageing, oxidative stress, disrupted proteostasis and other stimuli may induce the misfolding and aggregation of proteins leading to ER stress. In order to resolve ER stress, adaptive response through UPR is activated. However, prolonged ER stress induces apoptosis affecting neurons and synaptic funcion, thus leading to neurogenerative diseases (adapted from Hetz et al., 2017 [21]).

Misfolded proteins may lose their physiological activity, acquire neurotoxicity and lead to chronic brain inflammation [19]. Neuronal cells are particularly sensitive to protein misfolding, thus, excessive misfolding and aggregation lead to disrupted function of synapses, apoptosis and selective neuronal death [20] (Figure 2).

**Figure 3:**
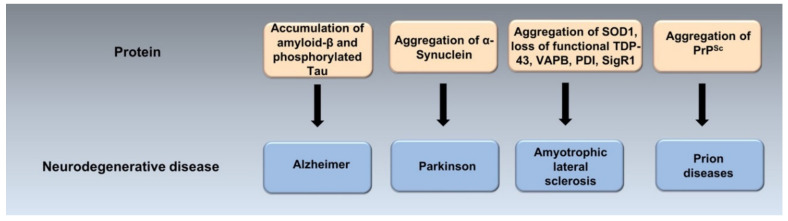
Protein misfolding and aggregation in the pathogenesis of neurodegenerative diseases. The accumulation of misfolded and aggregated particular proteins may play a crucial role in the pathogenesis of neurodegenrative diseases called protein misfolding disoders (PMDs). PMDs include Alzheimer’s (AD), Parkinson’s (PD), Amyotrophic lateral sclerosis (ALS) and prion diseases, among others. They are caused by misfolding and aggregation of amyloid-β and p-tau in AD, α-Synuclein in PD, SOD-1, TDP-43, VAPB, PDI, SigR1 in ALS and PrP^Sc^ in prion diseases.

ER stress mediates abnormal neuronal death in psychiatric diseases, such as schizophrenia, depression and post-traumatic stress disorder (PTSD) [21]. Evidence from transgenic animal models, neuropathological and genetic studies suggests that many neurodegenerative diseases are caused by the misfolding, aggregation and accumulation in the brain of an underlying protein (Figure 3) [22].

Accumulating evidence suggests that UPR mediators are directly involved in the physiopathology of protein misfolding disorders (PMDs) [23], thus ER stress markers have been described in neurodegenerative diseases. Interestingly, in patients’ brains affected with PMDs, ER stress markers often co-localize with protein aggregates [24,25]. Different UPR markers such as p-IRE1, active ATF6α, p-PERK and p-eIF2α, BiP and CHOP were found in patients with neurodegenerative diseases including Alzheimer’s [26], Parkinson’s [27,28], Amyloid lateral sclerosis (ALS) [29], prion diseases [30] among others [31]. p-PERK and p-eIF2α were detected in post mortem brain tissues of patients with different neurodegenerative diseases and this was associated with the accumulation of misfolded and aggregated protein [23,32]. BiP, phosphorylated forms of PERK, IRE1α and eIF2α were found in Alzheimer’s disease neurons and substantia nigra of Parkinson’s disease patients [30]. Furthermore, UPR markers were found to be overexpressed in spinal cord samples of ALS patients, in striatum, parietal cortex and caudate putamen of Prion disease patients [30].

Neuronal protein aggregates lose their function leading to the development of degenerative disorders. The accumulation of protein aggregate generates ER stress, followed by the activation of UPR. First, an adaptive response will start to alleviate the ER protein overload and preserve neuronal viability. Second, if ER stress persists, the ER stress-mediated cell death program will lead to neuronal loss. Intriguingly, each aggregated protein has a distinct mechanism of action, so different molecular mechanisms contribute to the imbalance of ER proteostasis and therefore neurodegeneration [21].

Finally, although misfolded proteins are implicated in the pathogenesis of neurodegenerative diseases, the exact relationship remains unclear. Additionally, whether ER stress plays a central role in the neurodegenerative diseases remains controversial. Some researchers have suggested that ER stress may play only a minor role in the mechanisms of neurodegeneration [33,34]. This review aims to discuss most recent findings relating PMDs, ER stress and UPR.

### 2.2. Interaction between ER Stress, Autophagy and Neurodegeneration

Autophagy is an important mechanism delivering damaged organelles, abnormal and misfolded proteins to the lysosome for degradation and leading to the recycling of the resulting macromolecules [35,36]. It plays an essential role in tissue remodeling, cell survival and regeneration [37]. Many studies in several model organisms have shown that the inactivation of autophagy genes leads to extensive cell death and to the presence of unnecessary components in the cytoplasm [37]. Neurons are vulnerable to impairments in autophagy; autophagic activity is important for maintenance of neuronal functioning since these cells are unable to dispose of aggregated proteins [38,39]. ER stress in chronic neurodegenerative disorders often stimulates autophagic activities, however, failure in clearing the aggregated proteins and the impairment of UPR or autophagy lead to the accumulation of misfolded proteins and the progression of neurodegeneration [40]. Impairment of autophagy via the knockdown of AuTophaGy(ATG)-related proteins ATG1, -2, -6, or -18 impeded synaptic development [41], impaired axonal integrity via altering microtubules stability and underlaid the onset and progression of various neurodegenerative disorders [42]. Autophagy-deficient mice showed neuronal defects and an impairment of autophagy was found to be associated with neurodegenerative diseases such as ALS, PD, AD and prion diseases [39,40].

ER stress may regulate autophagy via the PERK/eIF2α/ATF4 signaling pathway [43]. It was shown that ATG5 and ATG7 connect autophagy with ER stress through PERK signaling [44]. In response to ER stress, ATF4 was reported to guide the induction of autophagy gene transcription such as Beclin-1 (*BECN1*), microtubule associated protein 1 light chain 3 beta (*MAP1LC3B*), *ATG5, ATG7* and *ATG12* [45]. The eIF2α-kinases, GCN2 and PERK, ATF4 and CHOP were found to increase the transcription of a set of genes implicated in the formation, elongation and function of the autophagosome [45]. eIF2α dephosphorylation inhibition accelerated human cell death in an ER stress and autophagy-dependent manner [46]. These findings show that PERK pathway is pivotal for autophagy.

Moreover, IRE1α was studied in the context of autophagy and neurodegenerative diseases. IRE1/XBP1 pathway may influence the pathogenesis of neurodegenerative diseases. XBP1s acts as a transcription factor activating Beclin-1, the mammalian ortholog of the yeast autophagy-related gene 6 (Atg6) [47]. In addition, XBP1-FoxO1 (Forkhead box protein O1) interaction regulates ER stress-induced autophagy [48]. Blocking IRE1 or ATG7 expression was associated with dopaminergic neuronal loss, progressive locomotor impairment, shorter lifespan and the progression of PD, these findings show that IRE1 pathway couples ER stress to autophagy-dependent neuron death [49]. An interplay between IRE1/XBP1s and IRE1/TRAF2 (TNF receptor-associated factor 2)/ASK1/JNK (c-Jun N-terminal kinase) with autophagy was found. A recent study showed that in a Parkinson’s-like neurological disorder, IRE1 signaling pathway mediated the activation of JNK signaling via the formation of the ASK1-TRAF2 complex which can initiate the autophagy [50].

The ATF6 pathway was shown to be involved in interferon-gamma (IFN-γ)-induced activation of death-associated kinase 1 (DAPK1), an important regulator of cell death and autophagy [51]. Additionally, ATF6-mediated upregulation of CHOP, XBP1 and GRP78 contributes to ATF6-induced autophagy [52]. Less attention has been paid to ATF6 pathway involvement in autophagy; thus, further investigation is needed on this topic.

Finally, increasing evidence has indicated that autophagy and ER-membrane communication at membrane contact sites are closely related to neurodegenerative disorders, such as PD, AD, and ALS [53]. Vacuole membrane protein 1 (VMP1), an ER-localized metazoan-specific protein, interacts with Beclin-1 and plays important roles in the formation of autophagosomes and communication between the ER and other organelles [54].

## 3. ER Stress in Neurodegenerative Diseases

### 3.1. Alzheimer’s Disease (AD)

#### 3.1.1. ER Stress in AD

AD is a devastating neurodegenerative disease, affecting more than 25 million individuals worldwide, and characterized by progressive decline of cognitive functions. At the neuronal level, it involves excitotoxicity, neuronal oxidative stress, deregulation of intracellular signaling pathways, synapse damage/loss [55] and neurodegeneration in different brain regions among others within frontal cortex, the hippocampus and the basal forebrain [56]. The mechanisms leading to AD are complex, related to changes in synaptic transmission, an altered calcium homeostasis, increased ER stress and a chronic state of neuroinflammation [57]. The neuropathological hallmarks of AD are the accumulation of hyperphosphorylated tau and extracellular plaques consisting of amyloid-β (Aβ) peptides [57,58]. While Tau is a stabilizer of neuronal microtubules, phosphorylated tau (p-tau) is not and tends to aggregate into intracellular neurofibrillary tangles [59]. The amyloid β-precursor protein (APP) is cleaved by the β-secretase (β-APP cleaving enzyme 1; BACE1) and γ-secretase complexes to generate Aβ peptides, insoluble plaques, fibrils or soluble and diffusible oligomers that are described as highly neurotoxic leading to neurodegeneration and apoptosis [60]. γ-secretase is a membrane-associated complex consisting of the following four different proteins: presenilin-1/2 (PS1/2), nicastrin, Aph1 and Pen2 [61]. The catalytically active site of γ-secretase resides within PS1/2. Mutations in *APP* and in the Presenilin’s, *PS1* and *PS2* are associated with increased aggregation of the Aβ peptides in the brain’s parenchyma through an increased overall production of all Aβ species (e.g., *APP* duplications or *APP* mutations located around the β cleavage site) or production of a more aggregated form of the Aβ peptide, leading to AD [62]. During AD, the continuous accumulation of Aβ or p-tau results in a drastic alteration of ER calcium homeostasis, abnormal protein folding and ER stress. Tau has been shown to block the ERAD pathway leading to the accumulation of misfolded proteins in the ER lumen [63]. Aβ oligomers interacts with neuronal N-methyl-D-aspartate receptors (NMDA-Rs) and induces the disruption of the cytosolic calcium balance, provoking ER stress-dependent cell death, synaptic depression and spine elimination [64]. Aβ peptides neurotoxicity is related to ER stress-mediated apoptosis associated with ASK1 and JNK activation [65].

Several studies have noted the occurrence of abnormal levels of ER stress in the human AD brain. Under mild ER stress, UPR has a proadaptive role. However, in long-termed ER stress, UPR-pro-apoptotic branch is activated, which is considered a possible cause of neurodegeneration. BiP, along with other chaperones such as Hsp72, and Hsp73, Grp94, PDI and calreticulin were found upregulated in cerebrospinal fluids and AD brains [26,66]. Phosphorylated forms of PERK and eIF2α are markedly increased in AD brains especially in the hippocampal pyramidal cells and the frontal cortex [32,67]. This phosphorylation can be activated by the accumulation of tau aggregates [26]. The activation of PERK was shown to be associated with increased expression of the transcription factor ATF4 and BACE1 [68], since their mRNAs contain upstream open reading frames allowing a higher translation rate after eIF2α phosphorylation [69]. PERK insufficiency was associated with a decrease in BACE1 expression, thus reduced levels of Aβ peptides and plaque burden in a mouse model of AD [69]. Treating post-mortem frontal cortex tissues with salubrinal, an inhibitor of p-eIF2α phosphatase PP1c, directly increased BACE1 and Aβ production in primary neurons [70]. The presence of Aβ oligomers in the hippocampus was associated with increased ATF4 expression in the axonal compartment; ATF4 is a key regulator of the neuronal plasticity and hippocampal-dependent long-term spatial memory [71].

Furthermore, a positive correlation between the progression of AD histopathology and the activation of IRE1 was detected in human brain tissue. Genetic ablation of the RNAse domain of IRE1 in the nervous system significantly reduced amyloid deposition, the content of Aβ oligomers, and the neuronal loss. It is worth noting that IRE1 deficiency restored the learning and memory capacity of AD mouse model [72]. Moreover, a polymorphism in the promoter of the transcription factor XBP1 was identified as a risk factor of Alzheimer’s disease [73]. Studies on AD transgenic animal models have shown that XBP1 is linked to ADAM10 transcriptional regulation; ADAM10 (ADAM Metallopeptidase Domain 10) is the main α-secretase that cleaves APP [74]. Furthermore, XBP1 can reduce the expression of BACE1, through HRD1, leading to the reduction in Aβ plaques [75] and can upregulate the expression of catalytic components of the ERAD machinery [76].

The role of ATF6 in AD has not been reported until recently where Du et al. found that in an AD mouse model, the expression of ATF6 was reduced. ATF6 was found to reduce the expression of APP to suppress the Aβ level, downregulate the promoter activity and expression of BACE1, and protect the retention of spatial memory in AD model mice [77].

On the other hand, as a consequence of prolonged ER stress, an elevation of pro-apoptotic UPR components has been found in AD brains, likely causing neurodegeneration. The main transcription factor linking ER stress to apoptosis, CHOP, was found upregulated in the brains of AD patients, along with downstream effectors such as caspase-12, and GADD34 [55]. ER stress-mediated CHOP activation plays a central role in the triggering of AD pathological hallmarks. Upregulation of CHOP generates ROS, oxidative stress, high level of Aβ oligomers, inflammation of the neurons and eventually neuronal cell death via apoptosis [78].

Finally, the involvement of UPR in AD is still considered debatable, since in the 5XFAD (familial Alzheimer’s disease) transgenic mice for example, having enhanced expression of APP and PS1, UPR was not activated. The expression of BiP, p-IRE1, p-eIF2α, ATF4 and CHOP was not significantly elevated as compared to the non-transgenic control mice, suggesting that ER stress might not be a specific hallmark of AD, or at least is not induced by overexpression of APP and PS1 [34]. Additional studies are needed to further confirm the involvement of UPR in AD.

#### 3.1.2. ER Stress, Neuroinflammation and AD

A positive feedback loop exists between ER stress and inflammation, with clear implications for neurodegeneration and AD [55]. The three pathways of the UPR increase the production and the release of pro-inflammatory mediators such as the transcription factor NF-κB, known to activate the transcription of TNFα, IL-1, IL-6 and IL-8. Activated NF-κB regulates the expression of specific genes, including isoforms of the nuclear proto-oncogene *SET,* known to be elevated and mislocalized in the neuronal cytoplasm in brains of AD and directly implicated in the pathogenesis of AD [79]. In this context, p-IRE1 binds to the protein TRAF-2 and recruits the IKK (IκB kinase) complex, leading to the phosphorylation of IκB and resulting in its degradation and NF-κB activation [80]. In addition, the TRAF2-IRE1 complex may also bind ASK-1 to activate the JNK pathway, promoting inflammatory signaling through the transcription factor known as AP-1. JNK-AP1 signaling pathway is responsible for Aβ-induced neuroinflammation in an Alzheimer’s affected brain [81]. P-PERK, via P-eIF2α, inhibits the expression of IκB leading to an additional activation of NF-κB [55]. Furthermore, in the mouse brain, ER stress-induced activation of JAK1/STAT3 via P-PERK leads to neuroinflammation [82]. PERK may directly phosphorylate and activate GSK-3β (glycogen synthase kinase 3β), leading to the hyper-phosphorylation of tau, increased Aβ generation and deficits in learning and memory accompanied with neurodegeneration [83,84]. The role of ATF6 in proinflammatory action in brain pathology is under reported. It was shown that ATF6 siRNA abrogated the ER stress-mediated pro-inflammatory through protein kinase B(Akt)/NFκB activation [65] and the CREBH (cyclic adenosine monophosphate (cAMP)-responsive element-binding protein H) transcription factor [55].

### 3.2. Parkinson’s Disease (PD)

PD is a progressive neurodegenerative disease mostly diagnosed at a late stage of progression. PD comes after Alzheimer’s disease as the second most prevalent neurodegenerative disease [85]; it mainly affects people over 40 with a universal prevalence of 2% in men and 1.3% in women [86,87,88,89]. Tremor, impaired balance, posture and motion are the main symptoms of PD, and which progress with the severity of the disease. PD cases are mainly sporadic, only 10–15% of cases are caused by mutations in a variety of genes including *SNCA/PARK1* (encoding α-synuclein (α-SYN)), Parkin (*PRKN*), Parkinson’s disease protein 9 (*PARK9/ATP13A2*), Parkinson’s disease protein 7 (*PARK7/DJ1*), leucine-rich repeat kinase 2 (*LRRK2*), PTEN-induced putative kinase 1 (*PINK1*) and among other genes [90].

PD is generally characterized by two major hallmarks: (1) selective loss of dopaminergic (DA) neurons in substantia nigra pars compacta (SNpc), the region of the brain implicated in movement and muscle contraction control, and (2) the accumulation of misfolded, aggregated alpha-synuclein (α-SYN) fibrils within neuronal somas called Lewy bodies (LBs) or dendrites and axons as Lewy neurites (LNs) [91]. α-SYN is highly expressed in the presynaptic terminals of the CNS neurons but can also be found in peripheral tissues and blood [92,93]. A53T, A30P and E46K mutations of the *SNCA* gene, duplication and triplications of the wild-type gene were identified as a hereditary cause of PD [94,95,96], they act as dominant variants inducing the disorder. α-SYN monomers tend to aggregate, β-sheet conformation elongates into insoluble protofibrils and fibrils [27]. Misfolded α-SYN spreads from cell-to-cell through interconnected circuits of the nervous system [97], induces neuronal damage and has been associated with many neurodegenerative diseases other than PD, referred as α-synucleinpathies. 

Accumulating evidence supports the role of ER stress as a principal player in α-SYN toxicity and mediated dopaminergic neurons cell death. Numerous studies have shown that α-SYN aggregates interact with BiP, leading to the activation of the UPR signaling pathway when it binds to misfolded proteins [98,99]. In human brain tissues, ER stress markers, such as phosphorylated PERK and phosphorylated eIF2α, were detected in neuromelanin containing dopaminergic neurons of the substantia nigra of PD patients but not in control cases’ tissues [100]. Moreover, α-SYN was found to be more abundant in ER/microsomes fractions of human and mice PD brain tissues as compared to non-PD controls [101]. In an A53T transgenic mouse model, α-synucleinopathy coincides with the induction of ER chaperones and abnormal UPR in pathologic neurons. This is supported by the increase in ER stress associated polyubiquitin chains accumulation and caspase-12 activation. Interestingly, administration of an ER stress inhibitor, salubrinal, significantly ameliorated the onset of α-synucleinopathy in A53T transgenic mice and in an adeno-associated virus-transduced rat model of *A53T*α*S*-dependent dopaminergic neurodegeneration [101]. It was found to protect against cell death and reduce A53T cytotoxicity showing that ER stress is directly related to α-SYN mediated cell death [102,103]. In differentiated rat sympathetic-like neuron cells (PC12), overexpression of A53T α-SYN led to elevated intracellular ROS activity and decreased proteasome activity at a first stage followed by ER stress activation as a final protective stage before the induction of a caspase-dependent cell death. ER stress activation was detected by the elevation of eIF2 phosphorylation and the upregulation of ER stress related genes (*GRP17* and *GADD153*).

In dopaminergic differentiated SH-SY5Y cells (SH-SY5Y+), glucose deprivation induced α-SYN/ER stress-dependent cell death. In this model, α-SYN acts as a stress sensor where glucose deprivation leads to its overexpression and aggregation resulting in its interaction with GRP78/BiP, and consequent activation of the PERK-dependent pathway and ATF4/CREB-2 transcription factors [98]. Overexpression of ATF4/CREB-2 was detected in the substantia nigra of SYN120 transgenic mice as compared to control mice [98]. The role of IRE1-XBP1 pathway in linking ER stress to PD is controversial. While in animals injected with 6-hydroxydopamine (PD-inducing neurotoxin), gene therapy using the active form of XBP1 was found to be neuroprotective [104]. Recently, in a PD model in the fruit fly *Drosophila melanogaster*, IRE1 activation was found to cause cell loss in photoreceptor neurons in an XBP1-independent manner, which can be prevented by the inhibition of autophagy (knockdown of *ATG* genes, *ATG7* or *ATG8B*) [49].

On the other hand, using a genome-wide overexpression screen approach in yeast, Cooper AA. and colleagues showed that α-SYN accumulation causes ER stress by inhibiting the ER-Golgi trafficking [105,106]. It was reported that this trafficking impairment is caused by either a direct interaction with the ras-associated binding 1 (RAB1) GTPase [105,107] or by ATF6. In fact, co-expression of RAB1 with α-SYN rescued the loss of dopaminergic neurons in the animal models of Drosophila, C. elegans and in primary rat midbrain neurons [105]. Moreover, α-SYN inhibited ATF6 activation via coat protein complex II (COPII)-mediated ER–Golgi transit induced upon ER stress, leading to the attenuation of its cytoprotective effect, and apoptosis [108].

Another proposed model of ER stress induction by α-SYN aggregation is through ER Ca^2+^ homeostasis destabilization. In fact, α-SYN aggregates activate ER calcium pump SERCA (Sarco/endoplasmic reticulum Ca2+-ATPase) in neurons, leading to an alteration of the calcium metabolism, ROS production and apoptosis [109]. In mice, knockout of the *CaBP-9k* gene, a calcium binding protein expressed in the cytosol of dopaminergic neurons in the brain, increased α-SYN in dopaminergic neurons and induced ER stress-mediated apoptosis in neurons. Treatment of CaBP-9k KO mice with TUDCA (tauroursodeoxycholic acid), a pharmacological ER stress inhibitor, restored the expression of ER stress markers and cleaved caspase-12 to normal levels [110].

### 3.3. Amyotrophic Lateral Sclerosis (ALS)

ALS is an adult neurodegenerative disease marked by the degeneration of motor neurons in the cortex, spinal cord and brain stem resulting in muscle atrophy, paralysis, weakness and spasticity [111]. Most people with ALS have the sporadic form. Only a small proportion of people, estimated at 5 to 10 percent, have a familial ALS (fALS).

Many genes have been identified as ALS disease-causative, such as protein disulfide isomerase (*PDI*) and the superoxide dismutase1 (*SOD1*), TAR DNA-binding protein (*TARDBP* or *TDP-43*), fused in sarcoma (*FUS/TLS*), chromosome 9 open reading frame 72 (*C9ORF72*), vesicle-associated protein-associated protein B (*VAPB*) and Sigma-1 receptor (*SIGR1*) among others [24,111]. These mutations are linked to alterations in mRNA metabolism, proteostasis and aggregation of the affected protein, leading to neurotoxicity and neuroinflammation, thus ALS [111,112]. Many groups supported the involvement of ER stress in the pathophysiology of ALS, both in patients and animal models [113,114,115,116]. The upregulation of UPR markers was detected in ALS patients [113,114,115,116].

Approximately 20% of familial ALS, and 1–2% of all cases are caused by mutations in the gene encoding *Sod1* [117]. *SOD1*-mutant female ALS patients had a better survival rate than males [118]. The involvement of this mutation in ALS is the most investigated in research. Transcriptional analysis of motor neurons derived from induced pluripotent stem cells (iPSCs) of patients carrying *Sod1* mutation demonstrated that ER stress was high and UPR markers were upregulated [119]. Lumbal spinal cord sections’ examination of G93A *SOD1* mice showed an upregulation of PERK, IRE1 α and ATF6 [119]. UPR markers BiP, calnexin and PDI were found co-localized with mutant SOD1 in post-mortem tissue [21], where it might sequester these vital chaperones [120]. Alterations in ER morphology have been found in ALS patients and *SOD1* mouse model, including fragmentation of the rough ER, irregular distension of cisternae and detachment of ribosomes [121]. Mutant SOD1 interacts with Derlin-1, a component of ERAD machinery, triggers ER stress through dysfunction of ERAD, activates ASK1 and motor neuron death [122]. Initial studies indicated that *Perk* haploinsufficiency accelerates experimental ALS in *Sod1* transgenic mice, these mice had a reduced capacity to turn down synthesis of misfolded SOD1, leading to an early overload of the UPR [123]. Furthermore, the genetic ablation of *GADD34* [124] and the inhibition of eIF2α phosphatase [125] delayed the disease onset and prolonged motor neuron survival. However, more recent studies performed on *SOD1* mice showed that neither *PERK* haploinsufficiency, nor genetic UPR enhancement via ablation of GADD34, is beneficial for mutant *SOD1*-induced motor neuron disease [126]. ATF4 ablation in mutant *SOD1* G85R mice led to the protection against ALS, these mice appeared more resistant to ALS than mutant mice expressing normal levels of ATF4, possibly because of the reduced levels of apoptosis components, such as CHOP [127]. The role of IRE1 in ALS was shown by studying the homeodomain interacting protein kinase 2 (HIPK2), an essential component of the IRE1-ASK1 apoptotic cascade activating JNK under ER stress [128]. In *SOD1* G93A mice, loss of HIPK2 was associated with delayed disease onset, reduced cell death in spinal motor neurons, and improved survival [128]. Furthermore, the ablation of *XBP1* led to reduced motor neuron death and aggregation of mutant SOD1, mainly due to a homeostatic link between the UPR and the autophagy pathway [114]. The role of ATF6 in ALS is under reported. However, it was found that ATF6 levels were elevated in a *SOD1* G93A mouse model, suggesting that they play an important role in the progression of ALS [129].

The ER chaperones PDI, have a broad protective role in ALS. In fact, an increased amount of PDI was detected in the cerebrospinal fluid and in the blood of ALS patients indicating that any dysfunction of this chaperone contributes to the ALS pathology and aids in diagnosis [130]. A total of nine PDIA1 missense variants and seven PDIA3 missense variants were identified in ALS patients. Single nucleotide polymorphisms (SNPs) in both genes were also found enriched in ALS cases [131]. PDI variants were found to induce motor defects associated with a disruption of motoneuron connectivity and impaired dendritic outgrowth in motoneuron cell culture models [132]. Additionally, the over-expression of ALS-linked mutant *TDP-43* induced ER stress pathways in neuroblastoma cells and an interaction between PDI and TDP-43 was found in transfected cell lysates and the spinal cords of mutant A315T *TDP-43* transgenic mice [133]. The phosphorylation of eIF2α was found to be abnormally upregulated by TDP-43 aggregates in Drosophila and therapeutic modulation of eIF2α-phosphorylation attenuated TDP-43 toxicity in ALS [134]. In ALS models, FUS, which is normally located in the nucleus, translocates to the cytoplasm and forms inclusions; this has been linked to ER stress. Mutant FUS also colocalized with PDI in human ALS lumbar spinal cords and mutant FUS-linked familial ALS tissues [135]. In addition, *C9ORF72* mutation, a common cause of familial ALS, was found to be associated with an activation of the UPR; this mutation is associated with a vulnerability of motor neurons to Ca^2+^-permeable AMPA receptor-mediated excitotoxicity (AMPAR) known to be dysregulated in lower motor neurons in all ALS cases [136,137]. Mutations in genes encoding for ER proteins have been also linked to familial and sporadic cases of ALS, such as the mutation E102Q in the ER chaperone gene *SIGR1*; this mutation was found to lead to ER stress-mediated defects in protein homeostasis and dysregulation of RNA-binding proteins [138]. Furthermore, the disruption of the vesicle-associated protein-associated protein B (VAPB) was associated to ALS. The mutant protein VAPB P56S affects intracellular Ca^2+^ storage, Ca^2+^ signaling capacities and inhibits ATF6 and XBP1 [139]. Patients with ALS-associated *VAPB* mutations present chronic ER stress, synaptic loss and cell death [140]. Finally, alteration of DNA methylation and histone modification has been reported in ALS [141]. However, the picture of the impact of epigenetic modifications on the clinical aspects of ALS is still not clear [142].

### 3.4. Prion Diseases

Prion diseases or transmissible spongiform encephalopathies (TSEs) are a family of rare progressive neurodegenerative disorders that occur in both humans and animals. They include Creutzfeldt–Jakob disease (CJD), Gertmann–Straussler–Sheinker syndrome and fatal familial insomnia in humans, scrapie in sheep and goat, bovine spongiform encephalopathy (BSE, more commonly known as “mad cow disease”) in cattle, and chronic wasting disease (CWD) in cervids. Although there are three different etiologies of prion diseases, infectious, sporadic and hereditary, however, they are all characterized by the formation of an abnormal folding of the cellular prion protein (PrP), leading to the development of an abnormal protease resistant form [143]. In this context, the main molecular event in the pathogenesis of prion diseases is the conversion of the normal cellular α-helical prion protein (termed PrP^C^) into the pathological, β-sheet-rich, misfolded form known as PrP^Sc^ (for scrapie associated PrP) [144]. PrP^Sc^ is a marker for prion infection [145]; its deposition and accumulation are not intrinsically toxic [146]. It was proposed that the accumulation of PrP^Sc^ might stabilize ER misfolded subtypes inducing toxicity through different signaling cascades such as caspase activation, ER stress, autophagy, and calcium dysregulation—features found in prion diseases [146,147]. ER stress plays a significant role in the pathogenesis of prion diseases, not only by leading to the accumulation of PrP^Sc^ levels but also by increasing the misfolded form of PrP^C^ susceptible to prion conversion [148,149,150,151].

The involvement of ER stress in prion diseases was shown by the upregulation of the ER chaperone Grp58/ERp57 (ER protein) in parallel with PrP^Sc^ in scarpie-infected mice [152], Grp78/BiP, Grp94, PDIA1 and Grp58/ERp57 in the cortex of patients affected with variant CJD and sporadic CJD [153], and the heat shock protein 70 (HSP70) in CJD patients and scarpie-infected rodents [154,155]. Prion infection of mice lacking HSP70 showed accelerated disease progression suggesting that HSP70 may play an important role in suppressing or delaying prion disease progression [154].

Mainly PERK but also IRE1α and ATF6 pathways were shown to be involved in the prion disease. The modulation of UPR signaling prevented neuronal loss and increased survival in prion-diseased mice [54]. Recently, astrocytes from prion-infected mice presented increased p-PERK levels and neuronal degeneration; however, the authors were not able to determine if PERK signaling was activated because of the accumulation of misfolded prion protein or not [156]. The PERK pathway was reported to be activated in the hippocampus of prion-infected mice and those overexpressing PrP^c^ [157]. PrP accumulation induced an increase in p-eIF2α, leading to a reduction in the expression of relevant synaptic proteins, synaptic failure and neuronal loss in prion-diseased mice [23,157] and CJD patients [143]. The modulation of astrocytic PERK-eIF2α signaling in mice with prion disease was profoundly neuroprotective [54]. Interestingly, targeting p-PERK signaling in astrocytes during prion disease was alone sufficient to prevent neuronal loss and prolong survival thus UPR over-activation is neuroprotective [54]. Furthermore, in prion infected animals, gene therapy to deliver eIF2α phosphatase [157], oral treatment with an inhibitor of PERK [158] and treatment with ISRIB [159], a compound that blocks the consequences of p-eIF2α, provided neuroprotection, attenuated disease progression and protected against prion disease. Additionally, prion infection in mice induced the splicing of XBP1 mRNA and the activation of the stress kinases JNK and ERK (extracellular signal-regulated kinase) through IRE1α [160,161]. In a cellular model of prion disease, *XBP1s* overexpression prevented PrP misfolding [149,162], whereas a dominant negative form of *XBP1* and *IRE1*α significantly increased PrP aggregation [149,162]. The neuroprotective activity of XBP1 in prion diseases was tested in mice lacking *XBP1* in the brain; these animals showed normal prion replication and neuropathology, suggesting that other UPR pathways may compensate the absence of *XBP1* [161]. Furthermore, in a cellular model of prion disease, the overexpression of ATF4 or an active mutant form of ATF6 prevented PrP aggregation [115]. Overall data suggest that prion diseases are associated with ER stress response, where the PERK pathway is mostly involved. Future studies are needed to investigate in depth the role of IRE1 and ATF6 in the pathogenesis of these diseases.

## 4. Therapeutic Approaches: Chemical Compounds Targeting the UPR Pathways

Targeting the UPR pathway may be a valuable therapeutic strategy to control ER stress response associated with disorders such as neurodegenerative diseases. Recently, many chemical compounds and small molecules capable of targeting the UPR through different molecular mechanisms have been identified and tested. A selection of some of the most widely used drugs as promising therapeutic candidates will be presented in this review.

### 4.1. Chemical Chaperones

The use of chemical chaperones is a promising approach to alleviate ER stress in a range of diseases. The antidiabetic compound azoramide and BIP/GRP78 inducer X (BiX) were found to improve ER protein-folding capability in multiple systems [163,164]. In addition, 4-Phenylbutyrate (4-PBA) and tauroursodeoxycholic acid (TUDCA) have shown therapeutic benefits for a wide variety of diseases, such as AD, ALS and diabetes [165,166]. Furthermore, 2-phenylimidazo[2,1-b]benzothiazole derivatives (IBTs) were recently identified as chemical chaperones in a cell-based high-throughput screen. It was shown that IBT21 binds to unfolded or misfolded proteins, inhibits protein aggregation and prevents cell death caused by induced ER stress [165].

### 4.2. GlaxoSmithKline(GSK) 2606414

GSK2606414 is the first-generation PERK inhibitor. In fact, this inhibitor is a promising treatment strategy against neurodegeneration [167]. The reduction in PERK expression by GSK2606414 improved neuronal excitability and cognitive function in young normal mice; the hippocampal memory in middle aged mice was restored to the normal performance levels observed in young individuals [67]. In an in vivo experimental model of Marinesco–Sjögren syndrome, the inhibition of PERK pathway via GSK2606414 delayed Purkinje cell degeneration, prolonged the asymptomatic phase and improved the motor performance during the symptomatic phase [168]. As mentioned earlier, PERK signaling is activated in PD patients and rodent models of the disease. The oral administration of GSK2606414 inhibited this pathway in the SNpc after experimental ER stress stimulation, improved motor performance and increased dopamine levels and the expression of synaptic proteins [169]. The treatment of a mouse model of prion disease with this inhibitor induced a repression of protein synthesis associated with decreased levels of p-PERK, p-eIF2α, ATF4 and CHOP, showing the neuroprotective effect of GSK2606414 [158,167]. Moreover, in the frontotemporal dementia disease, the high levels of p-PERK, p-eIF2α and ATF4 were reduced after orally treating transgenic mouse model with GSK2606414 [170]. Recently, it was found that this compound is a potential therapy of cancer since it inhibits KIT, a type III receptor tyrosine kinase (RTK), known to activate many signal transduction pathways including RAS/ERK (Extracellular Signal-Regulated Kinase), PI3K/AKT or PKB (Phosphoinositide 3-kinase/ Protein kinase B), phospholipase C, JAK/STAT (Janus kinase/signal transducers and activators of transcription), and Src kinase pathways [171]. However, it has been reported that GSK2606414 may cause side effects related to pancreatic toxicity [169] maybe due to the extent of UPR inhibition, weight loss and hyperglycemia [158].

### 4.3. ISRIB

ISRIB, a small molecule which reverses the phosphorylation of eIF2α [172], inhibits the downstream targets of all eIF2α kinases including ATF4, CHOP, and GADD34, but it does not induce significant translational changes [167,173]. ISRIB has a promising therapeutic potential in vivo, it was found to enhance cognitive memory processes in brain-injured mice without inducing side effects [174]. In prion-diseased mice, the inhibition of the PERK pathway using GSK2606414 was profoundly neuroprotective but toxic to the secretory tissues. However, the treatment with ISRIB was neuroprotective without inducing any adverse effect on the pancreas, but it only partially restored global protein translation, as compared with GSK2606414 [159]. Recently, in an ALS rodent model, the inhibition of PERK signaling with ISRIB, but not with GSK2606414, significantly enhanced the neuronal survival via a partial inhibition of the translation imposed by PERK and a reduction in IRE1-dependent signaling [175]. In addition to neurodegenerative diseases, ISRIB was recently found to inhibit stress response signaling in the aging lung epithelium leading to decreased apoptosis and reduced recruitment of pathogenic monocyte-derived alveolar macrophages [176]. Moreover, the inhibition of PERK pathway by ISRIB reduced ATF4 and CHOP and two other eIF2α kinases: GCN2 (general control nonderepressible 2) and HRI (heme-regulated inhibitor) [177]. Finally, despite its neuroprotective effect, ISRIB cannot be used against human neurodegenerative diseases because of its insolubility [178].

### 4.4. Guanabenz

Guanabenz is an alpha-2-adrenergic receptor agonist safely used to treat hypertension. It is a small molecule that inhibits GADD34-mediated dephosphorylation of p-eIF2α, leading to increased p-eIF2α levels, modulates the synthesis of proteins and increases chaperone synthesis resulting in an adaptive response under ER stress conditions in human cells [179]. Guanabenz rescued motoneurons from misfolding protein stress both in in vitro and in vivo ALS models [167]. In an ALS transgenic mouse model, Guanabenz treatment prolonged the early phase of disease and survival as compared to untreated transgenic mice [180].

### 4.5. Sephin1

In mouse models, Sephin1, a derivative of guanabenz, was shown to inhibit eIF2α dephosphorylation via the inhibition of GADD34, as well as inhibit the global protein translation recovery, prevent stress-induced damage in cells and delay PMD development, such as Charcot-Marie-Tooth 1B, in ALS mice models [181]. However, Crespillo-Casado et al. found that in mammalian integrated stress response (ISR) deficient cells, Sephin1’s ability to suppress neurodegeneration was not due to the inhibition of eIF2α dephosphorylation but maybe due to its ability to attenuate IRE1 branch [182]. Finally, it was recently shown that intraperitoneal treatment with Sephin1 reduced PrPSc levels and ER stress-induced PrP aggregates, and prolonged survival of prion-infected mice [147].

### 4.6. Trazodone Hydrochloride and Dibenzoylmethane

Trazodone hydrochloride and dibenzoylmethane were identified by the National Institute of Neurological Disorders and Stroke (NINDS) small-molecule library screening performed on 1040 drugs. Both drugs reversed p-eIF2α mediated translational attenuation and were neuroprotective in two mouse models of neurodegeneration [178]. In prion-diseased mice, both drugs restored memory deficits, abrogated development of neurological signs, prevented neurodegeneration and significantly prolonged survival. Furthermore, in an animal model of tauopathy-FTD (frontotemporal dementia), these compounds rescued memory deficits and hippocampal atrophy [178]. Finally, both compounds were not toxic to the pancreas [178].

### 4.7. Salubrinal

Salubrinal acts as an activator of UPR branches, raises BiP levels, selectively inhibits eIF2α dephosphorylation, resulting in increased levels of p-eIF2α [183], interferes with death/survival-related signaling pathways such as ATF4 or ASK1 [184,185] and attenuates neuronal apoptosis [183]. Salubrinal may promote amyloidogenesis thus plays a key role in the mechanisms leading to AD development and progression [70]. Moreover, it was found to be effective in several models of PD. In A53T mutant mice, salubrinal reduced the accumulation of α-SYN in the ER, extended the life span, and delayed the onset of motor dysfunction [101]. In an A53T rat model, it was found to prevent neuronal loss and alleviate the symptoms [101]. In prion mice models, administration of salubrinal exacerbated neurodegeneration and reduced survival time [157].

### 4.8. Kinase Inhibiting RNase Attenuators (KIRA)

KIRA are aldehyde derivatives which bind to the active site of IRE1 by interacting with the lysine 309, target the RNase activity leading to IRE1 inhibition and the blockage of XBP1 splicing [186]. Recently, it was found that KIRA inhibitors interfere with IRE1 face-to-face dimer formation thus disabling the activation of the RNase domain [187]. KIRA may be a promising compound to treat diseases involving the activation of IRE1 pathway such as neurodegenerative diseases.

### 4.9. N-[2-hydroxy-5-methylphenyl)-3-phenylpropanamide

N-[2-hydroxy-5-methylphenyl)-3-phenylpropanamide is a small molecule known to activate the ATF6 arm of the UPR [188]. The activation of ATF6 was found to ameliorate disease-associated imbalances in ER proteostasis and function [189]. N-(2-hydroxy-5-methylphenyl)-3-phenylpropanamide was found to reduce the aggregation of amyloid disease-associated proteins in relevant cell models [188]. This treatment activated endogenous ATF6 through an increased processing by S1P and S2P [188], and through the modifications of a subset of ER proteins, including multiple PDIs [189].

## 5. Conclusions

ER stress plays an important role in many neurodegenerative disorders characterized by the accumulation of misfolded proteins and aggregates, such as AD, PD, ALS and prion diseases, among others. However, the involvement of the UPR and the mechanisms by which ER stress contributes to the pathogenesis remain not fully clear and may have contrasting and even opposing effects. This may be caused by the crosstalk between ER stress, UPR, energy control, global proteostasis and neuroinflammation. As discussed earlier, evidence from in vivo and in vitro neurodegenerative disease models showed that the accumulation of particular misfolded proteins associated with the disease leads to neuronal and synaptic dysfunction. Targeting UPR pathways to treat a range of neurodegenerative disorders is promising. Full understanding of the signaling pathways and physiological functions of UPR-related molecules will help to develop novel therapeutics. Identifying new drugs that can interfere with UPR signaling and target ER stress associated genes in different animal and cell models is crucial and will provide solid knowledge regarding the involvement of the UPR in PMD progression.

## Abbreviations

4-PBA	4-Phenylbutyrate
AD	Alzheimer’s disease
ADAM10	A disintegrin and metalloprotease domain 10
ALS	Amyloid lateral sclerosis
AMPAR	AMPA (α-amino-3-hydroxy-5-methyl-4-isoxazolepropionic acid) receptor
APP	Amyloid β-precursor protein
ASK1	Apoptotic-signaling kinase-1
ATF4	Activating transcription factor 4
ATF6	Activating transcription factor 6
ATG	AuTophaGy(ATG)-related proteins
Aβ	Amyloid-β
BACE1	β-APP cleaving enzyme 1
BECN1	Beclin-1
BiX	BIP/GRP78 inducer X
BSE	Bovine spongiform encephalopathy
C9orf72	Chromosome 9 open reading frame 72
CaBP-9k	Calbindin-D9K
Ca^2+^	Calcium
CHOP	C/EBPα-homologous protein
CJD	Creutzfeldt–Jakob disease
CNS	Central nervous system
COPII	Coat protein complex II
CRAC	Ca^2+^-release-activated Ca^2+^
CREBH	Cyclic adenosine monophosphate (cAMP)-responsive element-binding protein H
CWD	Chronic wasting disease
DAPK1	Death-associated kinase 1
eIF2α	Eukaryotic translation initiation factor α
ER	Endoplasmic reticulum
ERAD	ER-associated degradation
ERK	Extracellular signal-regulated kinase
ERMES	ER-mitochondria encounter structures
ERp	ER protein
FoxO1	Forkhead box protein O1
FTD	Frontotemporal dementia
FUS	Fused in sarcoma
GADD34	Growth arrest and DNA-damage-inducible 34
GCN2	General control nonderepressible 2
GRP17	Glycine rich protein 17
GRP78	78 KDa glucose-regulated protein
GSK-3β	Glycogen Synthase Kinase 3β
HD	Huntington disease
Herp	Homocysteine-inducible ER stress protein
HIPK2	Homeodomain interacting protein kinase 2
HRD1	HMG-CoA reductase degradation protein 1
HRI	Heme-regulated inhibitor
Hsp	Heat shock proteins
IBTs	2-phenylimidazo[2,1-b]benzothiazole
IFN-γ	Interferon-gamma
IKK	IκB kinase
IL	Interleukin
IP3R	IP3 receptor
iPSCs	Induced pluripotent stem cells
IRE	Inositol-requiring transmembrane kinase/endoribonuclease
ISRIB	Integrated stress response inhibitor
JAK/STAT JNK	Janus kinase/signal transducers and activators of transcription Jun-N-terminal kinase
KIRA	Kinase inhibiting RNase attenuators
LBs	Lewy bodies
LNs	Lewy neurites
LRRK2	Leucine-rich repeat kinase 2
MAM	Mitochondria-associated ER membrane
MAP1LC3B	Microtubule associated protein 1 light chain 3 beta
Mfn2	Mitochondrial fusion protein mitofusin 2
NE	Nuclear envelope
NF-κB	Nuclear factor-κB
NINDS	National Institute of Neurological Disorders and Stroke
NMDA-Rs	N-methyl-d-aspartate receptors
Osh	Oxysterol-binding homology
p38 MAPK	p38 mitogen-activated protein kinase
PARK	Parkinson’s disease protein
PC12	Pheochromocytoma of the rat adrenal medulla
PD	Parkinson’s disease
PDI	Protein disulfide isomerase
PE	Phosphatidylethanolamine
PERK	PKR-like ER kinase
PI3K/AKT PI4P	Phosphoinositide 3-kinase/ Protein kinase B Phosphatidylinositol 4-phosphate
PINK1	PTEN-induced putative kinase 1
PM	Plasma membrane
PMDs	Protein misfolding disorders
Pp1	Protein phosphatase 1
PRKN	Parkin
PrP	Prion protein
PS	Phosphatidylserine
PS1/2	Presenilin-1/2 (PS1/2)
PTSD	Post-traumatic stress disorder
RAB1	Ras-associated binding 1
RIDD	Regulated IRE1-dependent decay
ROS	Reactive oxygen species
RTK	Receptor tyrosine kinase
S1P	Site 1 protease
S2P	Site 2 protease
SERCA	Sarco/endoplasmic reticulum Ca^2+^-ATPase
Sigr1	Sigma-1 receptor
SNCA	Synuclein Alpha gene
SNpc	Substantia nigra pars compacta
SNPs	Single nucleotide polymorphisms
SOCE	Store operated Ca^2+^ entry
SOD1	Superoxide dismutase1
STIM	Stromal-interacting molecule
TARDBP or TDP-43	TAR DNA-binding protein
TNF	Tumor necrosis factor
TRAF2	TNF receptor-associated factor 2
TSE	Transmissible spongiform encephalopathies
TUDCA	Tauroursodeoxycholic acid
uORF	Upstream open reading frame
UPR	Unfolded protein response
VAP VAPB	Vesicle-associated membrane protein-associated protein Vesicle-associated membrane protein-associated protein B
VDAC1	Voltage-dependent anion selective channel protein 1
VMP1	Vacuole membrane protein 1
WT	Wild type
XBP1	X-box binding protein 1
α-SYN	α-synuclein
